# Erratum for Hsu et al., “Evaluating the susceptibility of various common cell lines and assessing inactivation conditions to Mpox virus”

**DOI:** 10.1128/spectrum.04220-25

**Published:** 2026-02-05

**Authors:** Shu-Chen Hsu, Ping-Cheng Liu, Shan-Ko Tsai, An-Yu Chen, Hui-Ping Tsai, Jun-Ren Sun, Ti-Yu Li, Pei-Yu Hsieh, Jyh-Yuan Yang, Tein-Yao Chang

**Affiliations:** 1 Graduate Institute of Medical Sciences, National Defense Medical University 662539, Taipei, Taiwan; 2 Institute of Preventive Medicine, National Defense Medical University 71548 , Taipei, Taiwan; 3 Graduate Institute of Applied Science and Technology, National Taiwan University of Science and Technology, Taipei, Taiwan; 4 Department of Cosmetic Science, Vanung University 63497, Taoyuan City, Taiwan; 5 Graduate Institute of Biodefense, National Defense Medical University 71548 , Taipei, Taiwan; 6 Department of Physiology and Biophysics, Graduate Institute of Physiology, National Defense Medical University 71548 , Taipei, Taiwan; 7 Division of Infectious Diseases and Tropical Medicine, Department of Internal Medicine, Tri-Service General Hospital, National Defense Medical University 71548 , Taipei, Taiwan; 8 Center for Diagnostics and Vaccine Development, Centers for Disease Control, Taipei, Taiwan

## ERRATUM

Volume 13, Issue 12, e00803-25, 2025, https://doi.org/10.1128/spectrum.00803-25. The affiliations and the affiliation numbers for each author should appear as shown in this erratum.

