# Supplementary material for: Evaluating the susceptibility of various common cell lines and assessing inactivation conditions to Mpox virus
Source: Microbiol Spectr. 2025 Nov 11;13(12):e00803-25. doi: 10.1128/spectrum.00803-25 (PMC12671154; doi:10.1128/spectrum.00803-25)

**Supplementary Figures**

**Supplementary Figure legend**

Figure S1. **Verification that Trizol does not impair filter performance.** (A) Amicon Ultra-4 filters were pre-treated and washed with either Trizol or PBS following the workflow described in the Methods. Subsequently, 1 mL of vaccinia virus (1×10^6^ pfu) was applied to each filter and concentrated to ~250 µL. The entire retentate (upper layer) was recovered and brought to 300 µL with E2 medium, while the flow-through (lower layer) collected beneath the membrane was retained separately. Both fractions (250 µL each) were serially diluted and quantified by plaque assay. (B) Plaque-assay results, expressed as mean ± SD of duplicates, are plotted as bar charts. Comparable viral titres in the retentate and the absence of detectable virus in the flow-through confirm that the filter maintained its integrity and retained infectious particles after Trizol exposure.

Figure S2. **Cytopathic effects at 96 hours post-infection following P1 and P2 Mpox virus infection in various cell lines.**

The mpox virus from different passages (P1 and P2) was used to infect multiple cell lines and incubated for 96 hours. The resulting CPE is shown for each cell type. "Mock" indicates uninfected controls. "P1-96h" refers to cells infected with first-passage Mpox virus and observed at 96 hours post-infection, while "P2-96h" refers to cells infected with Mpox virus derived from P1 and cultured in the same cell line for an additional 96 hours.

Fig. S1A


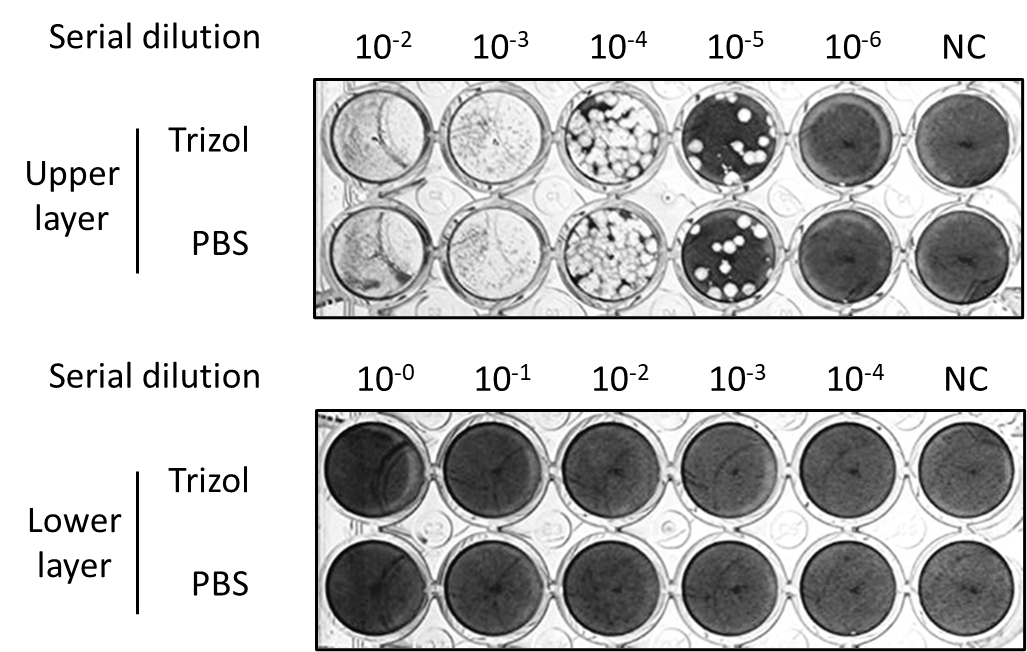


Fig. S1B


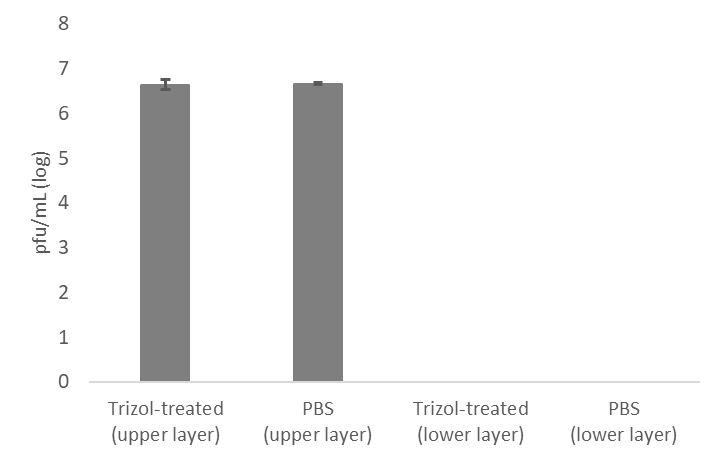


Fig. S2


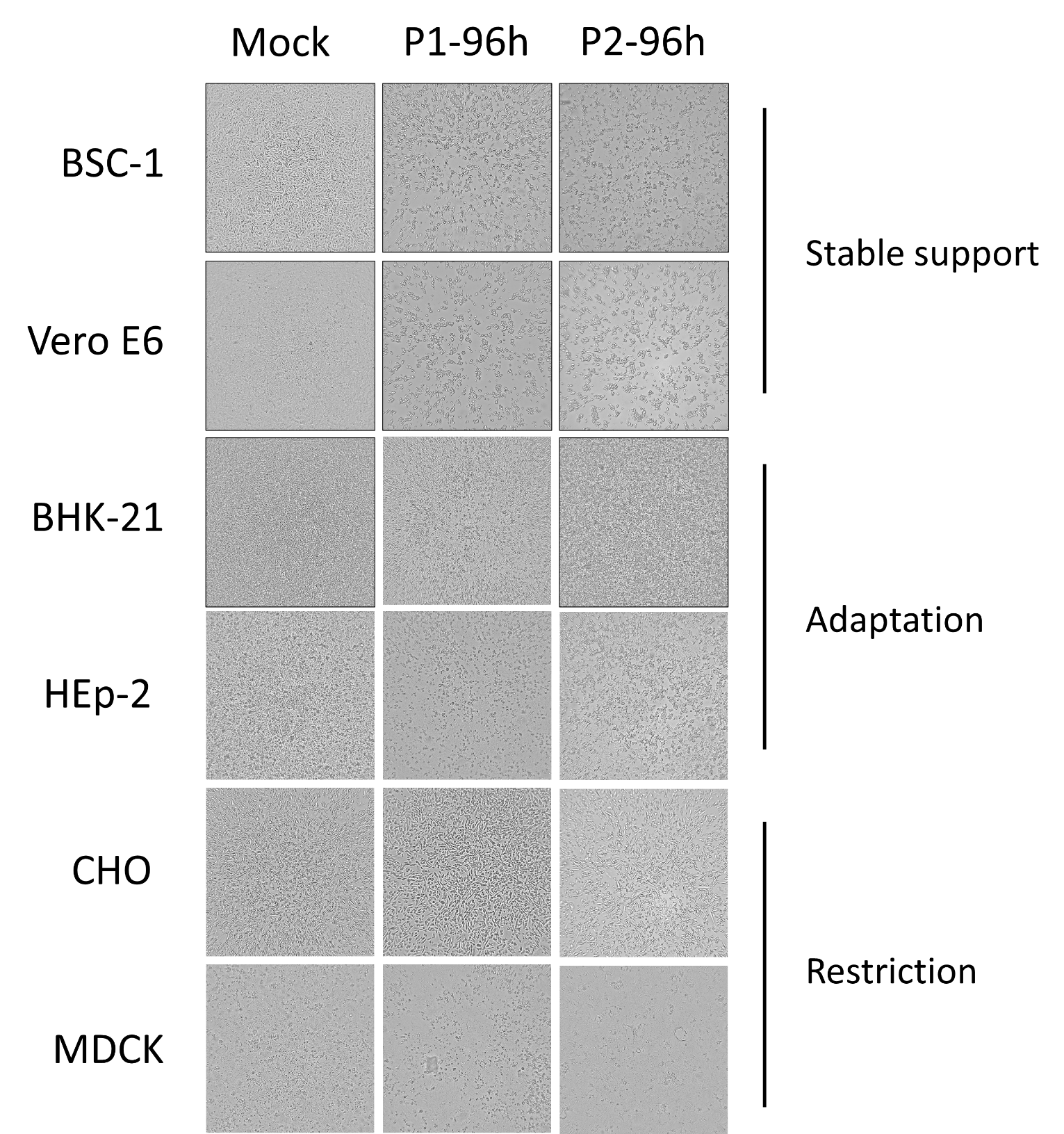

Supplement: Supplemental figures — Figures S1 and S2. [file spectrum.00803-25-s0001.docx]
